# Supplementary figures and images for: Functional and structural consequences of epithelial cell invasion by Bordetella pertussis adenylate cyclase toxin
Source: PLoS One. 2020 May 11;15(5):e0228606. doi: 10.1371/journal.pone.0228606 (PMC7213728; doi:10.1371/journal.pone.0228606)

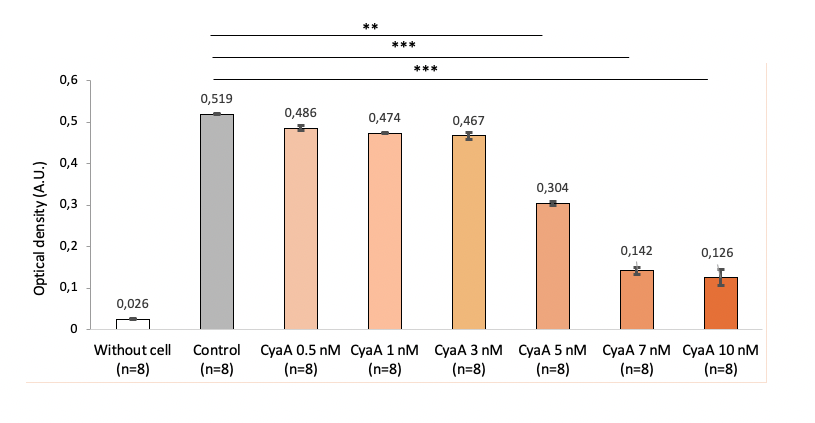

Supplement: S1 Fig — A549 cells were grown in monolayer to 90% of confluence and then incubated for 60 min with the indicated concentrations of CyaA. MTT was then added at 0.25 μg/ml and cells were further incubated 4 hrs at 37°C. The medium was removed and replaced by 200 μL of DMSO and the optical density at 550nm was recorded on a microplate reader. Control corresponds to cells incubated in similar conditions without CyaA. Error bars are ± SEM; * p ≤ 0.05; ** p ≤ 0.01; *** p ≤ 0.001. These data show that the viability of A549 cells is not significantly affected when they are exposed during 1 hr to CyaA concentrations lower than 3nM, while it is drastically reduced at CyaA concentrations above 5nM. (TIF) [file pone.0228606.s001.tif]

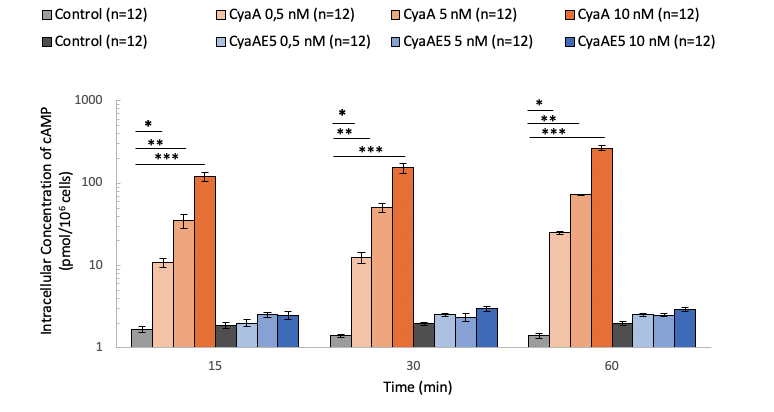

Supplement: S2 Fig — Intracellular cAMP is measured by ELISA assay in A549 cells exposed to CyaA or to CyaAE5, a CyaA variant lacking enzymatic activity, at concentrations 0.5; 5 and 10nM and for 15, 30, and 60 min (n = 12 wells). Control conditions correspond to cells incubated without toxin. Error bars are ± SEM; * p ≤ 0.05; ** p ≤ 0.01; *** p ≤ 0.001. These data show that even the lowest CyaA concentration (0.5nM) triggers a large increase in intracellular cAMP, that can be observed at the shortest exposure time (15 min) while very high cAMP levels can be reached observed at higher CyaA concentrations. As expected, no significant changes in intracellular cAMP levels are observed when cells are incubated with the enzymatically inactive toxin, CyaAE5. (TIF) [file pone.0228606.s002.tif]

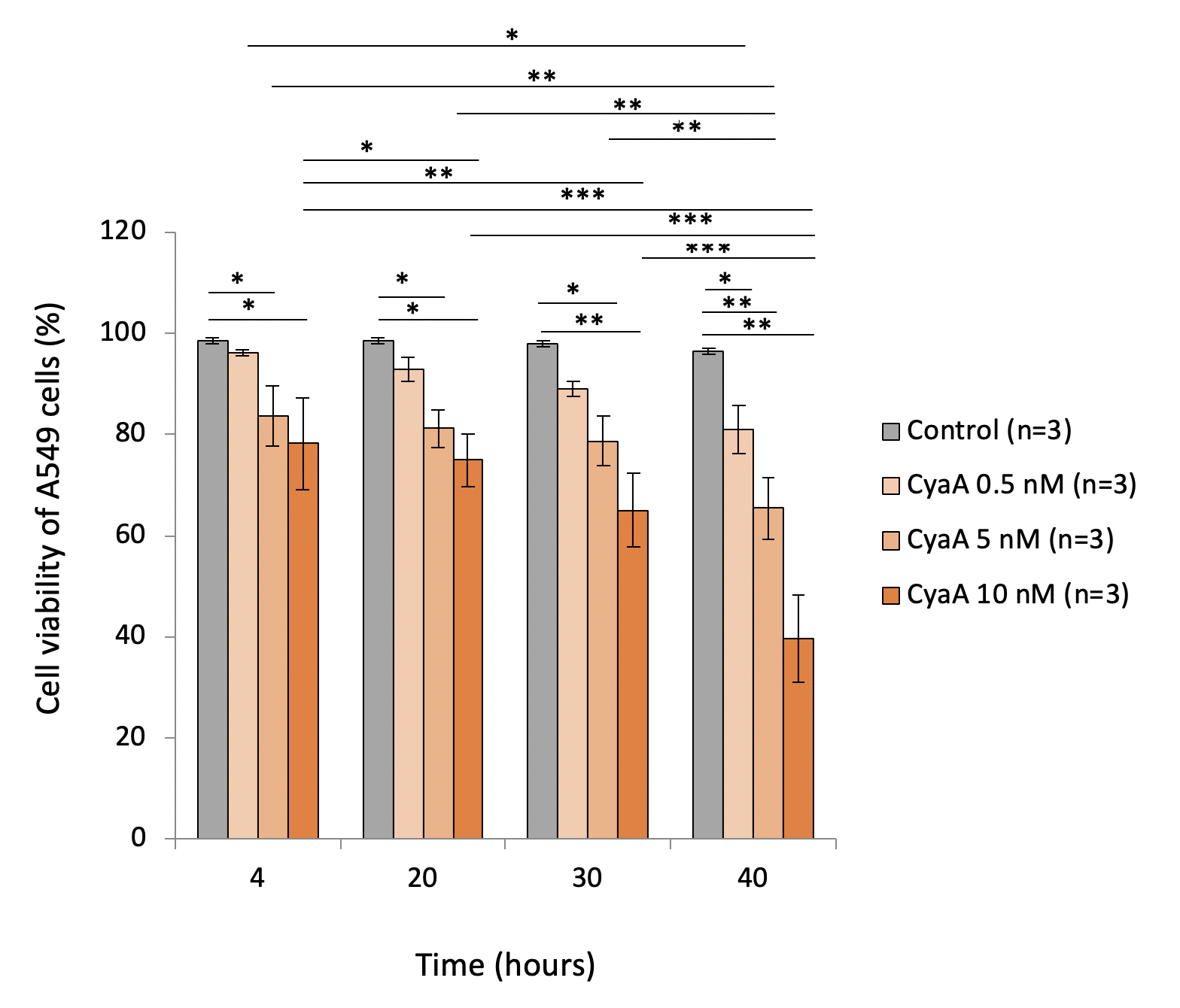

Supplement: S3 Fig — Viability assays performed by Trypan blue over 40 hrs on A549 cells in control conditions and after 1 hr of exposure time to different CyaA concentrations (0.5, 5 and 10 nM) (n = 3 wells). The test durations (4, 20, 30, 40 hrs) correspond to the times used for migration-repair experiments. The bar graph shows that the cell viability decreases with increasing CyaA concentration as well as with increasing test duration in many cases. * p ≤ 0.05; ** p ≤ 0.01; *** p ≤ 0.001. (TIF) [file pone.0228606.s003.tif]
