# Supplementary material for: Functional and structural consequences of epithelial cell invasion by Bordetella pertussis adenylate cyclase toxin
Source: PLoS One. 2020 May 11;15(5):e0228606. doi: 10.1371/journal.pone.0228606 (PMC7213728; doi:10.1371/journal.pone.0228606)
Supplement: Attachment — Submitted filename: Response to reviewers-01042020.pdf [file pone.0228606.s004.pdf]

We thank the reviewers for their assessments and comments which helped us to improve the quality of the manuscript. Here are our detailed answers to their questions.

***Reviewer #1:*** In the manuscript "Functional and structural consequences of epithelial cell invasion by Bordetella pertussis adenylate cyclase toxin" the authors describe major effects of CyaA on epithelial cell structure and function. This work builds incrementally on earlier data to show that these cells, when treated with CyaA, also display altered cell morphology, cytoskeletal stiffness and impaired wound healing capacity. The manuscript is clearly written, logically presented and nicely demonstrates the dramatic effect of CyaA intoxication on epithelial cells, a cell type not typically associated with CyaA-mediated pathologies. However, the authors should be careful not to attribute CyaA-mediated effects solely to elevated cAMP in those studies in which a control, demonstrating the cAMP-dependence, is not included. Major and minor comments provided below.

Major comments:

1. Experiments performed in Fig 7 could be improved with the addition of cytochalasin D to the CyaA treated cells to demonstrate the actin dependence of the CyaA-mediated effects

**Our answer**: Both CytoD and CyaA alter actin cytoskeleton structure integrity.

- In terms of cell morphology, CytoD and CyaA toxin both result in morphological changes exhibiting the same tendencies [see Fig. 6A and 6B for CyaA in A549 and see Fig. 4 of Ujihara *et al*. (J. Physiol. Sci. 58: 499-506, 2008) for CytoD effects in fibroblasts]:
    - Cell spreading area decreases significantly during both CyaA and CytoD treatments.
    - Cell height increases significantly during CyaA/CytoD treatment.
- In terms of cell mechanical properties, treatments by CytoD on the one hand and by CyaA on the other hand result in opposite evolutions (see Fig. 7A and 7B):
    - CytoD induces a decrease in CSK stiffness.
    - CyaA intoxication results in an increase in CSK stiffness.

Such differences between CytoD and CyaA effects reveal the high specificity of the CyaA-induced changes in cell morphology and mechanics. It is likely due to differences in the biological processes induced by the chemical drug (CytoD) and the pathogenic toxin (CyaA):

- CytoD alters both the deep and the cortical components of the CSK but the deep component is particularly modified (see Laurent *et al*., Annals of Biomedical Engineering, 2003, ref. [39] in the manuscript).
- By contrast, CyaA exposure alters preferentially the cortical CSK component as shown (i) by present data in Fig. 2 which reveals that the cortical actin CSK is more significantly affected by the increase in CyaA concentration than the dense actin CSK and (ii) by previous data on AFM (Atomic force Microscope) showing an increase in measured surface Young modulus (see Angely *et al*., Biol. Cell, 2017, ref. [16] in the manuscript).

The deterioration in actomyosin coupling processes (also synonymous of loss in intracellular tension) is most likely predominant after CytoD treatment while it might be marginal in the case of CyaA exposure (up to a certain extent because it probably depends on CyaA concentration). The moderate increase in CSK stiffness with increasing CyaA concentrations (see MTC experiments, Fig. 7 of the manuscript) as well as the weak correlation between cell stiffness and CyaA concentration (AFM experiments, Fig. 3C in Angely *et al*. (Biol. Cell, 2017, ref [16] of the manuscript) might reflect concomitant and opposite effects occurring during CyaA exposure. However, because the two techniques (MTC and AFM) are not strictly similar in terms of their responsiveness to CSK stiffness (directions of efforts and deformations differ), their sensitivity to changes in CyaA concentration may vary:

- the cell stiffness increase measured by MTC becomes significant at the two highest CyaA concentrations tested (5 and 10 nM in Fig. 7) and not at the lowest one.
- the changes in cell stiffness measured by AFM are more significant at the lowest CyaA concentrations tested (0.5 nM) and not at the highest ones. Interestingly, substituting CyaAE5 for CyaA at the lowest concentration abolishes the observed increase in cell stiffness.

For all aforementioned reasons, we believe that mixing the effects of CytoD and CyaA would lead to somehow confusing results. Each treatment (i.e., CytoD or CyaA) has by itself complicated effects and we were not sure to extract pertinent information by mixing both. This is why we did not test the effect of CytoD after each CyaA exposure. However, we did test the effect of CytoD in control conditions to assess CSK attachment (as shown in Figs 7A and 7B). Moreover, both CytoD and CyaA treatments affect cell adhesion (hence the reported cell morphological changes) and it would become highly difficult to understand adhesion effects when mixing them (see Angely *et al*., 2017 and Ujihara *et al*., 2008). The discrepancies between CytoD and CyaA effects are now underlined at the end of the 8$^{th}$ paragraph of the Results section.

***Reviewer #1:***

2. In earlier work, this group demonstrated that CyaA reduces the viability of A549 cells within 15 min of exposure to 10 nM CyaA, while a non-significant reduction was observed at 60 min with 0.5 nM CyaA. And, in Fig S1 CyaA is again demonstrated to reduce cell viability. The results presented in Fig 8 represent cells treated for 60 min with CyaA and measurements are displayed from 4 hours. In order to be consistent with the wound repair parameters, it would be good to include the cell viability as assessed at similar time points to those used in Fig 8. There is presumably significant loss in cell viability in treated cells in this assay. The authors should consider that a reduction in wound healing may not be solely attributable to a disrupted actin CSK. Loss in cell viability should be mentioned as a possible alternative mechanism or loss in cell viability should be controlled for in the experimental design.

**Our answer**: The reviewer is perfectly right: there is a significant decay in cell viability as CyaA concentration increases as shown in the new figure S3. In particular, there is a very significant loss of cell viability when A549 are exposed to the two highest CyaA concentrations tested (5 and 10 nM). This loss could indeed explain in part the huge decrease in wound repair observed

in these conditions. Yet, at the lowest CyaA concentration (0.5 nM), the decay of cell viability is marginal (except maybe at 40 hrs (lowest significance)) while the repaired area is significantly reduced for all durations considered up to 40 hrs (new Figure 8B). This confirms that CyaA-induced CSK alterations is a predominant factor of the degradation of migration-repair process. We added a comment in the Results section to highlight this important aspect (last paragraph).

***Reviewer #1:***

3. In the conclusions, it is stated that “the large increase in intracellular cAMP elicits various structural and functional modifications of the cells, including cell rounding, weakening of adhesions, remodeling of actin structures and of cytoskeleton stiffness” and that this “leads to a drastic inhibition of cell migration and wound repair capabilities”. However, in this manuscript, only the actions of CyaA on wound repair were proven to be cAMP-dependent, as the impact of CyaA was ablated when an enzymatically inactive CyaAE5 was added in place of active CyaA. In order to attribute the actions of CyaA to elevated cAMP levels for the remaining parameters, the authors must repeat these studies using CyaAE5 or with treatment targeting the reduction of cAMP e.g. Rp-8-Br-cAMPs

**Our answer**: Indeed, we did not systematically test as a control the enzymatically inactive CyaAE5 in the various assays, mainly because CyaAE5 had no detectable effects on cell viability, cell migration, or other cell phenotypes as measured here and in our prior article (Angely *et al*. Biol. Cell., 2017, ref [16] of the manuscript) on the A549 cells as well as on various other cell types (see for example refs [70], [71], [72] and reviewed in ref. [1]). We agree with the reviewer that it would be interesting to introduce the CyaAE5 controls and we had planned to repeat some measurements – at least with the highest CyaAE5 concentrations – just before the closure of our research sites, currently for an unknown time.

Yet, we can strongly infer that the structural and functional modifications of the cells upon exposure to CyaA as reported here are directly triggered by accumulation of intracellular cAMP as we clearly document the large and rapid increase in this second messenger upon addition of even small doses of CyaA (with no detectable changes with CyaAE5). The lack of any toxicity or effects on cell phenotype as well as on cell migration and wound repair (even upon prolonged time frame) observed in the presence of CyaAE5 – as compared to the drastic effects of wild-type CyaA – strongly supports the idea that CyaA in the tested conditions is mainly acting via cAMP-dependent signaling pathways.

Taking account of the pertinent caution raised by the reviewer, we modified our wording in the conclusion to avoid any over-interpretation of our data (line 415):

“In summary, we show here that the CyaA toxin is able to efficiently target alveolar epithelial cells where it triggers a rapid and large increase in intracellular cAMP. CyaA elicits various structural and functional modifications of the cells, including cell rounding, weakening of adhesions, remodeling of actin structures and of cytoskeleton stiffness.“

***Reviewer #1:***

Minor comments:

1. In the materials and methods, the description of A549 culture is extensive and may be reduced

**Our answer**: We removed 3 sentences in the description of A549 culture.

2. spell out the LQ amino acids when first described

**Our answer**: LQ has been replaced by “Leucine and Glutamine (LQ)”

3. provide first description for “ACD”

**Our answer**: ACD has been replaced by “Adenylate cyclase domain (ACD)“

4. provide first description for “SVF”

**Our answer**: FBS has been replaced by Fetal bovine serum (FBS)

5. Provide abbreviation for CSK at first usage (line 131 not line 240)

**Our answer**: The abbreviation CSK has been added to the title “**Actin Cytoskeleton (CSK) and Focal Adhesion Staining“**

6. Line 156, provide information on how cells were selected for structural analysis e.g. 5 random fields etc.

**Our answer**: The text has been modified as follows: “To analyze cellular images of cells selected by random fields, the mean level of….” Line 148

7. Section “Measure of CSK stiffness by MTC” identify the instrument used for this method

**Our answer**: The title has been modified to provide the full name of the technique.

8. Figure 8 could be improved by the addition of images of the end point of the wound healing assay; demonstrating a healed zone for control cells vs minimal healing by the CyaA-treated cells.

**Our answer**: Images of the wound healing assays have been added to the new Fig. 8 and displayed in Fig. 8A in the revised version. Legend of Fig. 8 has been modified accordingly.

9. In support of a cAMP-mediated role in impaired wound repair, it may be worth mentioning that A549 cells treated with cAMP-increasing molecules (forskolin, isoproterenol, dibutyryl cAMP) display reduced cell migration and wound healing (See RGS19 upregulates Nm23-H1/2 metastasis suppressors by transcriptional activation via the cAMP/PKA/CREB pathway)

**Our answer**: We thank the reviewer for this interesting comment. We added a new paragraph in the Discussion ($7^{th}$ paragraph) including 6 new references.

10. A549 cells possess some phenotypic plasticity, it could be interesting to determine whether high cAMP levels alter the cell differentiation state. For example, if it drives the cell away from a proliferative and reparative alveolar type 2 epithelial phenotype and towards a more quiescent AEC type 1 or perhaps even a neuroendocrine-like cell (high cAMP levels promote neuroendocrine differentiation in A549 cells, reference: Effect of A549 neuroendocrine differentiation on cytotoxic immune response)

**Our answer**: The reviewer raises an interesting point as indeed A549 cells can be differentiated. However, this process happens usually on a much longer time frame (several days to a week). In Mendieta *et al*. (*Endocrine Connections* (2018) 7,791–802 Effect of A549 neuroendocrine differentiation on cytotoxic immune response), the effects of neuroendocrine factors on the communication between the immune system and neoplastic cells is studied up to 120 hrs, which is far above the duration of present experiments. In this study, A549 cells were induced to neuroendocrine differentiation phenotype (A549NED) using cAMP-elevating agents and exhibited change in morphology in addition to changes in cell proliferation but this does not correspond to present experimental conditions. The same remark is valid for the study by Kondo *et al.* (*Stem Cells International*, Vol. 2015, Article ID 165867, Differential Regulation of Gene Expression of Alveolar Epithelial Cell Markers in Human Lung) in which the plasticity in gene expression of alveolar differentiation markers is studied in derived A549 clones up to 120 hrs.

By contrast, in the present study, the structural and functional consequences of CyaA intoxication are followed on a much shorter time frame (from hours up to a day and half) which is too short to expect a strong change in differentiation phenotype.

***Reviewer #2:*** Manuscript by Angely et al. describes structural and functional effects of Bordetella adenylate cyclase toxin on cytoskeleton of A549 alveolar epithelial cells. The main message of manuscript is far from being novel, nevertheless the manuscript is technically sound, and seems to merit PlosOne publication criteria.

The only major concern is the cytotoxicity of 5nM and 10 nM CyaA (Suppl.Fig.1) and the use of these CyaA concentrations in the other assays in addition to 0.5nM. One may then ask whether there is actually an effect of CyaA on cytoskeleton stiffness (Fig7) or whether the effect on cell migration is so dramatic (Fig8).

**Our answer**: We agree with the reviewer: the decrease in cell viability upon prolonged incubation with high CyaA concentrations (5 and 10 nM) likely explains in large part the huge decrease in wound repair observed in these conditions. We added a comment at the end of the “Results section” (and a new supplementary Figure S3) to highlight this important aspect. Yet, an interesting point from a physiological perspective is the effect of CyaA on wound repair at low concentration (0.5 nM) that might be reached locally during the infection. At this concentration, no significant cytotoxicity was observed.

Concerning the effect of viability alteration (cell death) on cytoskeleton stiffness measurements, we would like to mention that, in principle, only living cells contribute to the MTC signal. Indeed, if cells lose their stiffness because there are dead, microbeads (if still attached to integrin receptors which is unlikely) turn instantaneously by 90° and their contribution to the MTC signal is no more visible. If beads are detached from the cells and are floating in the medium, they do not contribute to the signal either. A good example is the effect of CytoD on CSK stiffness: CSK stiffness is significantly decreased (about 50%) meaning that bead rotation is dramatically increased while cell structure is deeply affected. Total cell destruction would lead to vanishing stiffness and internal tension, i.e., a 90° bead rotation. The fact that only living cells contribute to the MTC signal explains that one could still measure a significant cytoskeleton stiffness increase even after 24 hrs of cell exposure to CyaA (Fig 8B).

To precise this point, we added few sentences at the end of the subsection “Measure of the CSK stiffness by Magnetic Twisting Cytometry (MTC)” in “Functional assessments” (Material and methods section).

Noteworthy, in a previous study of our team published in *Biol. Cell* (Angely et al. 2017), we used Atomic Force Microscopy along with a new multiple force spectroscopy method to test the effect of CyaA on early adhesion sites. Only results obtained at the lowest CyaA concentration were published but we did also test adhesion at the two highest CyaA concentrations (5 and 10nM). These results were not published because cell debris altered the shape of the force-distance curves (from which we extract the (apical) cell stiffness). Thanks to the principle of the MTC technique, the contribution of dead cells can be ignored as explained in the previous paragraph.

Minor comment: Authors may want to explain better the rationale behind using manganese in their experiments.

**Our answer**: The idea behind the use of manganese is the reversibility of the intoxication in the cell cultures even though after CyaA exposure. The goal is to reactivate adhesion and then spreading via the integrins expressed at the cell membrane of A549 cells, knowing that adhesion and spreading is markedly affected by exposure to the toxin. Present study and our previous study on the alteration of development of early adhesion sites into mature focal adhesion show that adhesion process is affected by the CyaA toxin. Because manganese has a high affinity for integrins, it is known to be a powerful activator of integrins. The 2nd paragraph of the Results section has been extended to better explain the rationale of using $Mn^{2+}$. Among the two references quoted, a more recent reference by Mould et al. replace the previous one:

- Chen J, Salas A, Springer TA. Bistable regulation integrin adhesiveness by a bipolar metal ion cluster. Nat Struct Biol. 2003;10(12):995-1001,
- and Mould AP, Askari JA, Barton S, Kline AD, McEwan PA, Craig SE, Humphries MJ. Integrin activation involves a conformational of change in the alpha 1 helix of the beta subunit A-domain. J Biol Chem. 2002;277(22):19800-5.

***Reviewer #3:*** The authors present a data concerning the effects of CyaA (ACT) toxin on epithelial cells. CyaA has been extensively characterized in regards to its effects on phagocytes and other immune cells. The manuscript shows data speaking to the structural effects of high cAMP on cells in vitro. Multiple assays are used to backup the findings. Overall the manuscript it easy to read and clear.

Major concerns

- In the abstract one sentence reads: "We also show that, at the low concentrations that may be found in vivo during B. pertussis infection, CyaA impairs the migration and wound healing capacities of the intoxicated alveolar epithelial cells." This reviewer does not believe that data are fully available to determine the exact amount of CyaA in vivo during infection. Some studies have shown staining with anti-CyaA. Overall it is not clear how much CyaA is in the respiratory tract of experimental animals or infected humans. The authors should refrain for over-extrapolation of the data and revise the statement in the abstract to read "We also show that, at the low concentrations (insert amounts)...." It is appropriate for the authors to discuss CyaA in vivo concentrations in the discussion.

**Our answer**: We fully agree with the Reviewer that the precise amounts of CyaA that might be produced in vivo during infection by bacteria remain largely an educated guess based on the data published by Eby *et al*. (2013), ref. [13] of the manuscript. These data are nevertheless the only ones presently available and of course given the large uncertainty of many parameters, one should indeed consider these values as a rough indication of the potential ranges of concentrations that might be reached in the respiratory tract, more than a precise and robust determination of the CyaA levels in the corresponding tissues.

It should be noted yet that these values are in good agreement also with the levels of CyaA secreted in in vitro cultures (up to 1 to 3 µg/ml) in particular when appropriate media containing BSA were used (Bellalou *et al*. Infect Immun. 1990;58(10):3242-7 ; Gonyar *et al*. Infect Immun. 2017;85(6):e00198-17).

From these data, one can estimate that, when the bacteria are adhering to the respiratory tract epithelium, the local concentrations of secreted toxin at the bacteria – epithelium interface or in the immediate surroundings could easily reach subnanomolar and up to nanomolar ranges. Obviously, achieving precise measurements of the real local concentrations of CyaA *in situ* remains a daunting challenge (actually, to our knowledge we are not aware of any precise measurements of in vivo concentrations of any bacterial toxins secreted by their pathogens during the course of infection).

We modified our wording accordingly in the abstract (last 2 sentences) and also added a comment at the end of the second paragraph of the discussion.
